# The burden of progressive fibrotic interstitial lung disease across the UK

**DOI:** 10.1183/13993003.00221-2021

**Published:** 2021-07-08

**Authors:** Thomas Simpson, Shaney L. Barratt, Paul Beirne, Nazia Chaudhuri, Anjali Crawshaw, Louise E. Crowley, Sophie Fletcher, Michael A. Gibbons, Philippa Hallchurch, Laura Horgan, Ieva Jakaityte, Thomas Lewis, Tom McLellan, Katherine J. Myall, Ryan Miller, David J.F. Smith, Stefan Stanel, Muhunthan Thillai, Fiona Thompson, Timothy Wallis, Zhe Wu, Philip L. Molyneaux, Alex G. West

**Affiliations:** 1 Dept of Respiratory Medicine, Guy's and St Thomas’ NHS Foundation Trust, London, UK; 2 Bristol Interstitial Lung Disease Service, North Bristol NHS trust, Southmead Hospital, Bristol, UK; 3 Academic Respiratory Unit, University of Bristol, Bristol, UK; 4 Leeds Interstitial Lung Disease Service, St James's University Hospital, Leeds, UK; 5 North West Lung Centre, Wythenshawe Hospital, Manchester, UK; 6 Birmingham Interstitial Lung Disease Unit, Queen Elizabeth Hospital Birmingham, University Hospitals Birmingham NHS Foundation Trust, Birmingham, UK; 7 University Hospitals Southampton NHS Foundation Trust, Southampton, UK; 8 South West Peninsula ILD Network, Royal Devon and Exeter Foundation NHS Trust, Exeter, UK; 9 Dept of Interstitial Lung Disease, Royal Papworth Hospital NHS Foundation Trust, Cambridge, UK; 10 Royal Brompton Hospital, London, UK; 11 National Heart and Lung Institute, Imperial College London, London, UK; 12 Contributed equally as last authors

## Abstract

While idiopathic pulmonary fibrosis (IPF) remains the exemplar progressive fibrotic lung disease, there remains a cohort of non-IPF fibrotic lung diseases (fILD) which adopt a similar clinical behaviour to IPF despite therapy [1]. This phenotypically related group of conditions, where progression of disease is similar to that seen in IPF, have recently been described as progressive fibrotic interstitial lung diseases (PF-ILD) [2]. Historically, treatments for these cases have been limited though given the phenotypic similarities many cases may have been given a multidisciplinary working diagnosis of IPF based on their disease behaviour [3]. The INBUILD trial broadened the scope of treatable fILD by demonstrating a significant benefit of Nintedanib in patients with fILD and progressive disease [4]. In response to this the European Commission approved an additional indication for nintedanib in adults for the treatment of PF-ILD in July 2020.

## Abstract 2

**Almost 15% of new referrals with non-IPF fibrotic ILD go on to develop a progressive fibrotic phenotype and would benefit from antifibrotic therapy** https://bit.ly/3uPhClN

*To the Editor*:

While idiopathic pulmonary fibrosis (IPF) remains the exemplar progressive fibrotic lung disease, there remains a cohort of non-IPF fibrotic lung diseases (fILD) which adopt a similar clinical behaviour to IPF despite therapy [1]. This phenotypically related group of conditions, where progression of disease is similar to that seen in IPF, have recently been described as progressive fibrotic interstitial lung diseases (PF-ILD) [2]. Historically, treatments for these cases have been limited though given the phenotypic similarities many cases may have been given a multidisciplinary working diagnosis of IPF based on their disease behaviour [3]. The INBUILD trial broadened the scope of treatable fILD by demonstrating a significant benefit of Nintedanib in patients with fILD and progressive disease [4]. In response to this the European Commission approved an additional indication for nintedanib in adults for the treatment of PF-ILD in July 2020.

While research interest grows in the progressive phenotype and debates about the optimal diagnostic criteria continue the incidence of patients with PF-ILD potentially eligible for treatment according to the criteria laid out in the INBUILD trial remains unclear. Previous attempts to estimate the proportion of fILD who develop a progressive fibrotic phenotype have either used estimates based on the disease behaviour of individual conditions [5], interviews with experts [6] or analysis of insurance claims [7]. This has resulted in estimates ranging from 18 to 40% of all fILD that will develop progressive disease. With the anticipated approval of therapeutic interventions for this cohort of patients worldwide, including in the UK, there is an urgent need to refine these estimates in a real-world population to enable appropriate service provision.

This retrospective, observational study therefore aimed to estimate the incidence of PF-ILD across England. Nine centres providing commissioned tertiary referral services for ILD were included. All new referrals seen for their first outpatient clinic appointment between August 1, 2017, and January 31, 2018, were assessed against the diagnostic criteria for PF-ILD laid out in the INBUILD trial [8] and, in particular, the criteria for progression: relative decline in forced vital capacity (FVC) % predicted ≥10%, or FVC decline ≥5% but <10%, combined with worsening respiratory symptoms, or FVC decline ≥5% but <10%, combined with radiological progression; or radiological progression with worsening respiratory symptoms. A full chart and imaging review was undertaken of all the subjects. Continuous variables are presented as mean±sd, and categorical variables as proportions. Time-to-event curves were calculated using the Kaplan–Meier method and compared with the use of the log-rank test.

A total of 2368 patients with ILD were assessed across the nine centres. 619 patients were diagnosed and managed as IPF and therefore excluded, leaving 1749 patients with fILD who were screened against the INBUILD criteria for progression, to identify cases of PF-ILD either at the first clinical review, or in the subsequent 2 years of follow up. In the cohort of patients at risk of developing PF-ILD the INBUILD criteria were met in 14.5% (253 out of 1749) of all new non-IPF fILD referrals despite standard therapy, with a range between these specialist ILD centres from 8.9% to 23.6% of total cases. The average time from referral to specialist centre to diagnosis of progressive phenotype was 311±273 days and, at the time of referral, 20% of patients demonstrated progressive disease (66 out of 253) despite standard therapy. Almost all patients received at least one immunosuppressive agent, with the majority receiving either oral or intravenous corticosteroids (96%). A number of second-line agents were employed with mycophenolate (46%) the most commonly used. Five of the subjects with PF-ILD received antifibrotic therapy on compassionate grounds.

The most common diagnoses associated with a PF-ILD phenotype were chronic hypersensitivity pneumonitis (84 (33.2%) out of 253), unclassifiable ILD (44 (17.3%) out of 253), connective tissue disease-associated ILDs including rheumatoid arthritis-associated ILD (42 (16.6%) out of 253) and non-specific interstitial pneumonitis (36 (14.2%) out of 253). In the PF-ILD cases, the mean age was 68±12.4 years and, interestingly, 53.4% of the cohort was female, compared with the well-recognised male predominance seen in IPF. This is likely driven by the significant female predominance in both chronic hypersensitivity pneumonitis and connective tissue disease which make up almost half of the PF-ILD cases.

Patients with progressive disease had a significantly higher mortality compared to those with non-progressive fILD (hazard ratio, 3.32; 95% confidence interval, 2.53–4.37; p=<2×10^−16^). Indeed, the survival of patients with PF-ILD was no different to the subjects with IPF (hazard ratio, 1.06; 95% CI 0.84–1.35; p=0.6) (figure 1).

**FIGURE 1:**
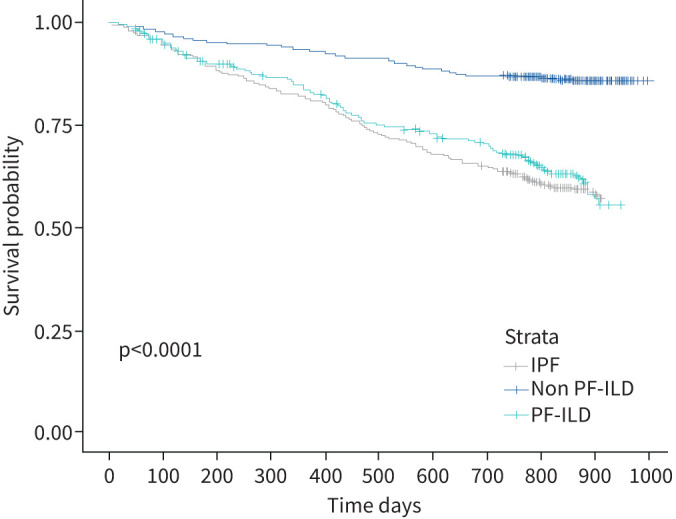
Kaplan–Meier curves comparing survival between patients with idiopathic pulmonary fibrosis (IPF), progressive fibrotic interstitial lung diseases (PF-ILD) and non-progressive non-IPF fibrotic lung diseases (non PF-ILD). Log-rank p test value is reported.

Of the progression events, the majority were driven by a measured drop in FVC, with more than half of patients (52.2%) experiencing a drop of ≥10%. A further quarter of patients (24.1%) were diagnosed with progressive disease on the basis of radiological and symptomatic progression alone without a spirometric deterioration. The remainder experienced a decline of FVC between 5 and 10% with either radiological (15.8%) or symptomatic (7.9%) progression.

The variations between centres and clinicians in diagnostic pathways, approaches to follow-up and definitions of progression has previously made it difficult to define and assess this cohort of patients. One of the strengths of our approach was the central collation and uniform application of the INBUILD inclusion criteria. However, this was done retrospectively and this is the main limitation of our study. While the INBUILD trial criteria are mostly objectively measurable phenomena, the definition of progressive symptoms may allow some biasing towards inclusion in those cases where spirometric progression was either not evidenced or not available, thus increasing the numbers of cases. Over a quarter of referrals received a final multidisciplinary team diagnosis of IPF, and this is often pragmatic and based on their clinical disease behaviour, to allow access to antifibrotic therapy. However, a patient's initial clinical and radiological features may have had more in keeping with a different ILD but with a PF-ILD phenotype. While all of the cases underwent local ILD multidisciplinary assessment, we did not undertake any central reassessment and therefore some cases of non-IPF PF-ILD may have been missed. Progression was only assessed at presentation and over a period of 2 years, as per the INBUILD screening criteria; however, we do know that progression may occur later during follow-up [9] and therefore some late progressors would not have been captured in this analysis. Our estimates therefore may be if anything an underestimate but importantly they reflect current clinical practice, which we aimed to capture.

This study represents a fair and balanced approach to assessing the incidence of objectively measurable and treatable PF-ILDs in the UK. A rate of 14.5% of new referrals with non-IPF ILD is less than that reported in previous studies however our methodology is likely to give a more accurate result than estimates based on extrapolation from general disease statistics, from physician-reported estimates prone to significant biases, or insurance claim processes also substantially prone to bias. This information has implication for workforce planning and the funding of anti-fibrotic therapy in the UK and beyond.

## Shareable PDF

10.1183/13993003.00221-2021.Shareable1 This one-page PDF can be shared freely online.
Shareable PDF ERJ-00221-2021.Shareable
