# Supplementary material for: The burden of progressive fibrotic interstitial lung disease across the UK
Source: Eur Respir J. 2021 Jul 8;58(1):2100221. doi: 10.1183/13993003.00221-2021 (PMC8264777; doi:10.1183/13993003.00221-2021)
Supplement: Supplementary file 1 [file ERJ-00221-2021.Shareable.pdf]

The burden of progressive fibrotic interstitial lung disease across the UK

SHAREABLE PDF

Thomas Simpson[1], Shaney L. Barratt [2,3], Paul Beirne[4], Nazia Chaudhuri[5], Anjali Crawshaw[6], Louise E. Crowley[6], Sophie Fletcher[7], Michael A. Gibbons[8], Philippa Hallchurch[2], Laura Horgan[4], Ieva Jakaityte[8], Thomas Lewis[2], Tom McLellan[9], Katherine J. Myall[1], Ryan Miller[8], David J.F. Smith[10], Stefan Stanel[5], Muhunthan Thillai [9], Fiona Thompson[7], Timothy Wallis[7], Zhe Wu [10,11], Philip L. Molyneaux [10,11,12] and Alex G. West [1,12]

[1]Dept of Respiratory Medicine, Guy's and St Thomas' NHS Foundation Trust, London, UK. [2]Bristol Interstitial Lung Disease Service, North Bristol NHS trust, Southmead Hospital, Bristol, UK. [3]Academic Respiratory Unit, University of Bristol, Bristol, UK. [4]Leeds Interstitial Lung Disease Service, St James's University Hospital, Leeds, UK. [5]North West Lung Centre, Wythenshawe Hospital, Manchester, UK. [6]Birmingham Interstitial Lung Disease Unit, Queen Elizabeth Hospital Birmingham, University Hospitals Birmingham NHS Foundation Trust, Birmingham, UK. [7]University Hospitals Southampton NHS Foundation Trust, Southampton, UK. [8]South West Peninsula ILD Network, Royal Devon and Exeter Foundation NHS Trust, Exeter, UK. [9]Dept of Interstitial Lung Disease, Royal Papworth Hospital NHS Foundation Trust, Cambridge, UK. [10]Royal Brompton Hospital, London, UK. [11]National Heart and Lung Institute, Imperial College London, London, UK. [12]Contributed equally as last authors.

Corresponding author: Philip L. Molyneaux (p.molyneaux@imperial.ac.uk)

Check for updates

Shareable abstract (@ERSpublications)
Almost 15% of new referrals with non-IPF fibrotic ILD go on to develop a progressive fibrotic phenotype and would benefit from antifibrotic therapy https://bit.ly/3uPhClN

Cite this article as: Simpson T, Barratt SL, Beirne P, *et al*. The burden of progressive fibrotic interstitial lung disease across the UK. *Eur Respir J* 2021; 58: 2100221 [DOI: 10.1183/13993003.00221-2021].

This single-page version can be shared freely online.

Copyright ©The authors 2021.

This version is distributed under the terms of the Creative Commons Attribution Licence 4.0.

Received: 19 Oct 2020
Accepted: 26 Feb 2021

To the Editor:

While idiopathic pulmonary fibrosis (IPF) remains the exemplar progressive fibrotic lung disease, there remains a cohort of non-IPF fibrotic lung diseases (fILD) which adopt a similar clinical behaviour to IPF despite therapy [1]. This phenotypically related group of conditions, where progression of disease is similar to that seen in IPF, have recently been described as progressive fibrotic interstitial lung diseases (PF-ILD) [2]. Historically, treatments for these cases have been limited though given the phenotypic similarities many cases may have been given a multidisciplinary working diagnosis of IPF based on their disease behaviour [3]. The INBUILD trial broadened the scope of treatable fILD by demonstrating a significant benefit of Nintedanib in patients with fILD and progressive disease [4]. In response to this the European Commission approved an additional indication for nintedanib in adults for the treatment of PF-ILD in July 2020.
